# Conventional ultrasonography enabled with augmented reality needle guidance for percutaneous kidney access: an innovative methodologies randomized controlled trial

**DOI:** 10.1097/JS9.0000000000002033

**Published:** 2024-08-08

**Authors:** Chaojie Xu, Aolin Li, Yiji Peng, Lin Li, Gengyan Xiong, Yu Fan, Zheng Zhao, Xin Li, Xiaochun Zhang, Yaoyao Zheng, Chi Zhang, Changning Lv, Xuesong Li, Gang Wang, Yan Xia, Pu Wang, Lin Yao

**Affiliations:** a Department of Urology, Peking University First Hospital, Beijing; b Department of Urology, The First Affiliated Hospital of Shenzhen University, Shenzhen Second People’s Hospital, Shenzhen; c Institute of Medical Photonics, Beijing Advanced Innovation Center for Biomedical Engineering, School of Biological Science and Medical Engineering, Beihang University, Beijing; d Weipeng (Suzhou) Medical Devices Co., Ltd, Suzhou, Jiangsu, People’s Republic of China

**Keywords:** augmented reality, image-guided surgery, kidney stone, percutaneous nephrolithotomy, surgical navigation, ultrasound-guided

## Abstract

**Importance:**

Successful needle puncture of the renal collecting system is a critical but difficult procedure in percutaneous nephrolithotomy (PCNL). Although fluoroscopy and ultrasound are the standard imaging techniques to guide puncture during PCNL, both have known limitations.

**Objective:**

To assess the feasibility and safety of a new navigation system for needle puncture in ultrasound-guided PCNL.

**Design:**

This study employed a single-center randomized controlled trial (RCT) design to assess the feasibility and safety of a new navigation system for needle puncture in ultrasound-guided PCNL. Conducted between May 2021 and November 2021, the trial utilized computer-generated random numbers for participant allocation to control for selection bias.

**Setting:**

The trial was executed at Department of Urology, Peking University First Hospital in Beijing, China, which serves as an academic medical center.

**Participants:**

All patients who met the inclusion criteria were randomly divided into two groups, with 29 patients in each group. One group underwent PCNL procedures using the new navigation system, while the control group underwent standard ultrasound-guided PCNL procedures. Included patients had renal pelvis or caliceal calculi larger than 2.0 cm in diameter or had multiple or staghorn stones. The puncture procedure was performed with the support of real-time ultrasound imaging and visual guidance displayed on the screen.

**Main Outcomes and measures:**

The primary outcome was system feasibility and puncture success rate. Secondary outcomes included puncture time, total surgical time, number of attempts, postprocedure complications, and 1-year and 3-year stone recurrence rates. Stone clearance was defined by postoperative CT. Descriptive statistics summarized patient demographics, stone size, and location. Independent samples *t*-tests analyzed puncture time and total surgical time. *χ* ^2^ or Fisher’s exact tests compared stone clearance, complications, socioeconomic status, renal hydronephrosis, stone location, race, and medical history. Linear regression examined the correlation between BMI and puncture time. Significance was set at *P*<0.05.

**Results:**

For all 58 patients undergoing PCNL, needle punctures of the renal collecting system were completed with a success rate of 100%. The average time from planning the puncture protocol to successful puncture was significantly shorter in the AcuSee guidance system group (3.12 min, range 0.2–6.88 min) compared to the standard ultrasound-guided group (7.58 min, range 5.41–10.68 min), representing a reduction of ~59%. The total surgical time was also shorter in the AcuSee group for patients with no and mild hydronephrosis (*P*<0.05). Complication rates were lower in the AcuSee group, with no major complications observed. However, three patients in the standard ultrasound-guided group have adverse effects after the PCNL procedure. The 1-year stone recurrence rate was significantly lower in the AcuSee group (3.4%) compared to the standard group (24.1%), and the 3-year recurrence rate was also lower (6.9% vs. 41.4%). Patient-specific factors such as BMI, renal morphology, and prior surgical history did not significantly affect the performance of the AcuSee system.

**Conclusions and relevance:**

The authors report the first clinical application of a new navigation system for needle puncture in ultrasound-guided PCNL. It has been demonstrated that it is feasible and safe compared to the standard ultrasound-guided group in percutaneous renal puncture. This technology provides intuitive and easy-to-use visual guidance, which may facilitate safe, accurate, and fast needle puncture of the kidney.

## Introduction

Highlights

- RCT confirms the new system’s feasibility.
- 100% puncture success rate achieved.
- Reduced puncture time by 4.46 min.
- No major complications in the test group.

Large kidney stones can have significant impacts on both patients and healthcare costs. If not treated promptly, large kidney stones can lead to complications such as urinary tract infections, renal obstruction, and potential renal failure, which increase the burden on both patients and healthcare systems. Surgical intervention is necessary for large kidney stones with a diameter greater than 2.0 cm or staghorn calculi, but these procedures carry risks of adverse events (AEs) including bleeding, infection, and sepsis. In the United States, the incidence of large kidney stones has nearly doubled over the past few decades^1–4^. Concurrently, healthcare costs associated with kidney stones have risen at an alarming rate, with the economic burden estimated to exceed $5 billion annually^5^. The average cost per patient undergoing PCNL is substantial, with ultrasound-guided PCNL procedures costing ~$5258.90 per case^6^. Therefore, ensuring patient safety and reducing healthcare costs are crucial in the management of large kidney stones.

Percutaneous nephrolithotomy (PCNL) is typically the first-choice treatment for patients with large kidney stones^1,7^. Among all the steps of PCNL, establishing an accurate and effective percutaneous tract to collecting system is a critical but challenging technique that has a significant impact on the clinical outcome^8,9^. Existing technologies, such as fluoroscopy and ultrasound, face several limitations. Fluoroscopy, while providing clear images, exposes both the patient and the surgeon to radiation, increasing the risk of radiation-related complications^10^. On the other hand, ultrasound guidance, though radiation-free, offers relatively low resolution and contrast. As a two-dimensional imaging modality, it makes the needle trajectory more difficult to observe and manipulate, potentially increasing the risk of organ injury and bleeding^11^.

Historically, needle guidance technologies have evolved significantly. Early techniques relied solely on tactile feedback and anatomical landmarks, which had high failure rates and complication risks. The introduction of imaging modalities like fluoroscopy and ultrasound in the late 20th century marked a major advancement, improving success rates and safety profiles. Ultrasound and fluoroscopy are currently the most widely used imaging guidance for PCNL^1^. Recent developments have focused on enhancing these modalities with technologies such as optical tracking and augmented reality to overcome existing limitations^12–15^.

To overcome these problems, a new navigation technology, the AcuSee Ultrasound-Assisting Surgical Guidance System (model AS-P1000, Weipeng Suzhou Medical, Suzhou, China), was developed with optical tracking and augmented reality as core technologies. The name ‘AcuSee’ is a combination of two elements: ‘Acu’ stands for ‘Accuracy’, emphasizing the precision of the system, and ‘See’, indicating the visual guidance aspect of the system. Together, ‘AcuSee’ represents a system that provides precise and accurate visual guidance for surgical navigation. This system obtains the positioning information of the ultrasound probe and the needle installed with optical tracking mounts to assist ultrasound-guided needle placement by visualizing the real-time position, orientation, and trajectory of the needle on ultrasound images. Other existing technologies, such as electromagnetic tracking systems and virtual reality enhancements, have attempted to address these complications. Electromagnetic systems^16–19^, while effective in maintaining needle visibility, suffer from interference issues and require additional in vivo components. Virtual reality systems^20^ provide detailed anatomical overlays but lack real-time adaptability and often involve complex setups. The AcuSee system offers significant improvements by integrating optical tracking and augmented reality, providing real-time, high-precision guidance without radiation exposure or the need for complex in vivo components.

We conducted a randomized controlled trial (RCT) to evaluate the feasibility and safety of the AcuSee guidance system in humans. Prior to this, rigorous in vitro testing on phantom models and in vivo testing on animals were conducted. These studies focused on evaluating the feasibility on phantom and animal models, providing a foundation for the current research. The in vitro testing assessed the accuracy and puncture time using the AcuSee system with an ultrasound machine on a gelatin phantom model. The results showed improved accuracy (distance to target: 1.31 mm vs. 3.03 mm) and efficiency (puncture time: 8.93 sec vs. 21.6 sec) for novice users. The in vivo testing demonstrated the feasibility of the AcuSee system in the PCNL workflow by successfully guiding multiple needle punctures of pig kidney calyxes by surgeons of varying experience levels, achieving a success rate of 80% and an average puncture time of 57 s. Preliminary results have demonstrated that the device can help inexperienced operators improve the accuracy and time efficiency of punctures on phantom models and validate the feasibility of the AcuSee system in the PCNL workflow. This trial marks the inaugural application of the AcuSee system in human subjects.

## Methods and patients

### Study design

We conducted a single-center RCT at Peking University First Hospital (PKUFH, Beijing, China), an academic medical center, to assess the feasibility and safety of the AcuSee guidance system for PCNL. This represents the first global RCT of this navigation system. From May 2021 to November 2021, a total of 58 patients with kidney stones who met the inclusion criteria participated in the study. The determination of this sample size was informed by our expectation for the puncture navigation technology. Specifically, the anticipated one-time successful puncture rate using this technology is 89% is not lower than 70%. Employing the PASS 15 software to compute the noninferiority test for two independent sample rates and setting parameters at α=0.025, ß=0.2, we deduced a required sample size of at least 29 cases per group.

To ensure the integrity of the study, using computer-generated random numbers, patients were evenly and randomly allocated into two distinct groups, tallying 29 patients each. The first group underwent PCNL procedures deploying the AcuSee guidance system. In contrast, the control group was subject to standard ultrasound-guided PCNL procedures. This meticulous randomization process ensured each patient was justly assigned to one of the two designated groups.

Preoperative mid-stage urine culture was performed in all cases. If a urinary tract infection was present, preoperative aggressive anti-infection treatment was performed^21^. Blood glucose was controlled at <10 mmol/l, and normal coagulation function was required in all patients. Patients were enrolled in this study after a routine preoperative evaluation. The stone size was measured at the maximum diameter on operative computed tomography (CT).

Informed consent was obtained from all eligible participants before the procedure. Institutional review board approval (2020keyan398) was obtained from PKUFH before starting the study.

### Patient selection criteria

#### Inclusion criteria

Participants must be at least 18 years of age and have either renal pelvis or caliceal calculi larger than 2.0 cm in diameter, multiple stones, or staghorn stones^22^. Furthermore, they should be diagnosed by physicians as requiring PCNL for stone removal and must provide informed consent to participate in the study.

#### Exclusion criteria

The study excludes patients with conditions such as horseshoe kidney, pelvic kidney, or pyonephrosis^1^. Those with contraindications for the surgery, a history of upper urinary tract urothelial carcinoma or coagulation dysfunction, or any condition preventing them from adhering to the treatment (e.g. mental disorders) are also excluded.

#### Removal criteria

Participants will be removed from the study if they do not meet the inclusion criteria, violate the study’s medication protocols, or fail to take prescribed medications impacting the outcome measures. Incomplete data that affects the assessment of efficacy and safety will also lead to removal.

#### Drop-out criteria

Cases will be dropped if the patient exits the study due to adverse reactions or ineffective treatment, or if the patient is lost to follow-up.

#### Termination criteria for participants

The study will be terminated for a participant if, from a medical standpoint, it is deemed necessary by the researcher or if the patient requests to stop the trial.

### Study endpoint definitions

#### Primary outcome

The primary endpoints were the feasibility and accuracy (in terms of the success rate of puncture) of the system for clinical use. We recorded the success rate of puncture (the percentage of all attempts that are successful).

#### Secondary outcomes

Secondary outcomes included puncture time, total surgical time, number of attempts, postprocedure complications, and 1-year and 3-year stone recurrence rates. We recorded the procedural time (defined as the time from the start of planning to the end of a successful puncture) and the number of puncture attempts undertaken (puncture was defined as each movement or redirection of the needle followed by a forward movement, with or without new skin penetration). Short-term effects within 7 days of PCNL were evaluated using the Clavien–Dindo surgical complication grading system to systematically categorize and record the occurrence of one or more complications^23^. Complications included displacement of the DJ tube after surgery, the need for treatment due to bleeding, and postoperative fever, among others. Complications were categorized as follows: Grade I: Minor complications requiring no or minimal intervention, such as mild postoperative fever; Grade II: Complications requiring pharmacological treatment, including the need for treatment due to bleeding and more severe postoperative fever; Grade III: Complications requiring surgical, endoscopic, or radiological intervention; Grade IV: Life-threatening complications requiring intensive care management; Grade V: Death related to the surgical procedure. Long-term effects were assessed primarily through the stone recurrence rates at 1-year and 3 years postoperation^24^.

The collected patient demographic data included sex, age, BMI, comorbidities (such as hypertension, diabetes, and previous kidney disease), stone size, stone location, duration of kidney disease, previous treatments or surgeries for kidney stones, lifestyle factors (such as smoking status, alcohol consumption, and dietary habits), socioeconomic status, and geographic distribution. Stone size was measured on preoperative CT and defined as the maximum size of the largest stone. The extent of hydronephrosis was also assessed. Stone clearance and complications were assessed. Stone clearance was defined as clinically insignificant stone fragments of ≤4 mm obtained by postoperative renal, ureteral, and bladder CT scans^25–28^.

### Device design

**Figure 1:**
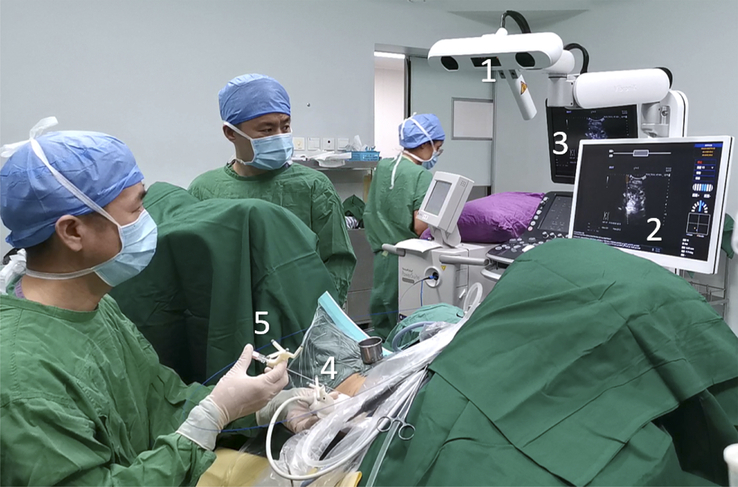
AcuSee system setup for puncture of percutaneous nephrolithotomy (PCNL) in a clinical setting. The setup consists of the following components: (1) a tracking module facing the operation site that detects the position and orientation of the ultrasound probe and puncture needle; (2) a touchscreen for displaying the ultrasound image and user interface; (3) an ultrasound machine connected to the AcuSee system with a video cable; (4) and (5) a tracking mount installed in each of the ultrasound probe and puncture needle to make them trackable; and (6) software running on a PC that process data collected from other modules, calculates guidance information and controls the system.

The AcuSee system consists of six primary modules, as shown in Figure 1:

1. *Tracking Module*, which provides infrared lighting, tracks the position and orientation of tracking mounts within the intended tracking volume and transmits the tracking result to the hosting personal computer (PC).
2. *PC Module*, which provides computing power and interfaces to interact with other modules and external systems (including the ultrasound system and operator), stores data, calculates, and illustrates the ultrasound images augmented with visualized guidance information.
3. *Mobile Cart Module*, which provides enclosure, mounting, and mobility for the system.
4. *Powering and Wiring Module*, which provides power supply, circuit protection, electrical protection, insulation, cooling, input/output interfaces, electrical connection, and start-up function.
5. *Accessories Module*, which provides tracking mounts for the ultrasound probe and needle/needle-like rigid devices to be detected and tracked by the system.
6. *Software*, which provides the user interface and backend programmes for system start-up, visualization of the guidance information, control of guidance, and system maintenance.

The process of using the AcuSee system is shown in Supplementary Video 1 (Supplemental Digital Content 1, http://links.lww.com/JS9/D277) and Supplementary Video 2 (Supplemental Digital Content 2, http://links.lww.com/JS9/D278). The system seamlessly connects to any existing ultrasound machine with a high-definition multimedia interface (HDMI) or video graphics array (VGA) video output port. Once connected, the system captures ultrasound images in real-time. At the core of the AcuSee system is the optical tracking module, which is mounted on the top of the device. This module projects infrared light into the tracking space, detecting the tracking mounts installed on both the ultrasound probe and the needle. The real-time tracking data collected by the optical tracking module is transmitted to the PC module. The software on the PC processes this data, combining it with preconfigured information to generate precise visual guidance. This guidance is then superimposed onto the ultrasound image stream, which is displayed on a high-definition touchscreen. The user can interact with the system through this touchscreen and an attached keyboard, allowing for intuitive control of the guidance information.

The AcuSee system introduces several key innovations: it enhances precision by providing real-time visual guidance, simplifies user interaction with an intuitive interface, and maintains compatibility with any standard ultrasound machine, eliminating the need for additional specialized equipment. This combination of features makes the AcuSee system a versatile and valuable tool for improving the accuracy and efficiency of ultrasound-guided procedures.

### Analysis of the operational principles of the AcuSee system

**Figure 2:**
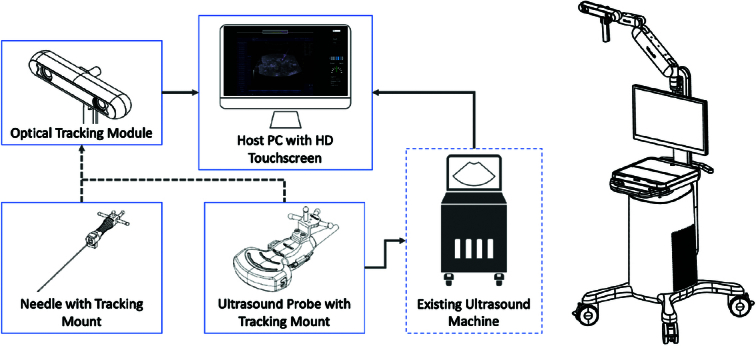
Schematic diagram illustrating the internal modules of the AcuSee system when integrated with an existing ultrasound imaging machine and its display. The system comprises four main modules: 1) Optical Tracking Module, 2) Host PC with HD Touchscreen, 3) Needle with Tracking Mount, and 4) Ultrasound Probe with Tracking Mount. The AcuSee system connects to the ultrasound imaging machine via a video cable to capture live ultrasound images. Infrared (IR) retro-reflective spheres on passive tracking mounts attached to the ultrasound probe and needle allow the optical tracking module to determine their three-dimensional positions. This information is used to project real-time guidance information, including position, trajectory, and offset distance and angle of the needle relative to the ultrasound imaging plane, onto the ultrasound image displayed on the HD touchscreen.

To illustrate the operational principles of the AcuSee system, we provide a schematic diagram (Fig. 2) describing its internal modules when used with an existing ultrasound imaging machine. The AcuSee system enhances the ultrasonic image of interventional needles or needle-like devices, such as biopsy, aspiration, or ablation needles, and predicts their future path on a display that also shows the ultrasound image. It is designed for procedures where ultrasound is currently used for visualization.

The AcuSee system connects to an existing ultrasound imaging system via a video cable, capturing live ultrasound images. Two passive tracking mounts with four infrared (IR) retro-reflective spheres each are attached to the ultrasound probe and the interventional needle, respectively. The optical tracking module tracks the three-dimensional (3D) positions of the IR spheres using computer stereo-vision methods and identifies these spheres within the defined tracking mounts. The AcuSee system calculates the spatial relationship between the needle and the ultrasound imaging plane based on the 3D positions of each tracking mount. This information, including the position, trajectory, offset distance, and angle of the needle relative to the ultrasound imaging plane, is superimposed on the real-time ultrasound image displayed on the HD touchscreen.

### Workflow

**Figure 3:**
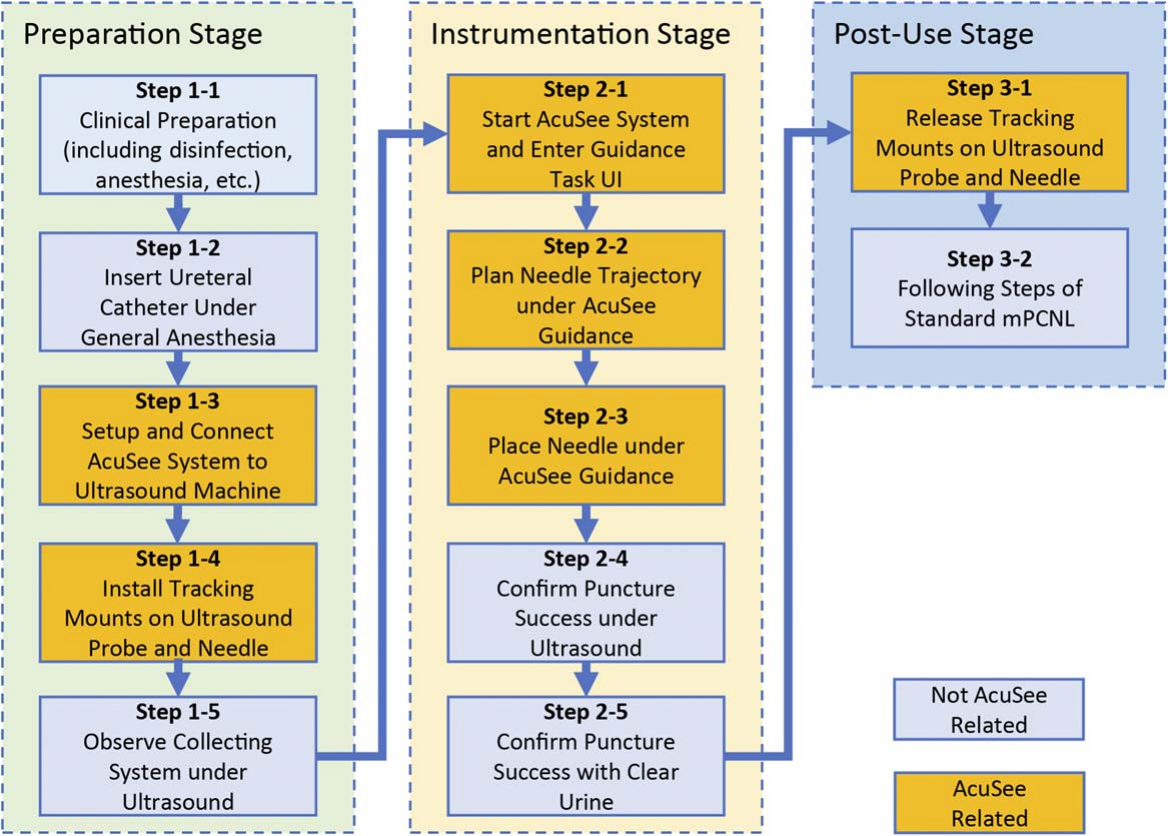
Workflow of using the AcuSee guidance system for percutaneous nephrolithotomy (PCNL).

**Figure 4:**
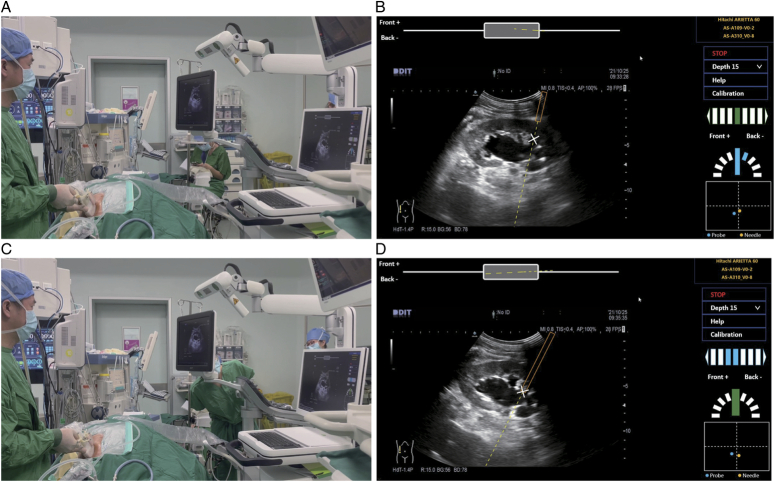
Under the guidance on needle trajectory and orientation displayed over the ultrasound image, the needle trajectory was planned for accessing the lower calyx (A, B) and puncture success was confirmed under ultrasound (C, D). In the bottom left area, an image window displays the ultrasound image overlaid with the needle trajectory in real-time, which consists of the part of the needle already within the image boundary (represented by an orange rectangular box) and its predicted trajectory (represented by a yellow dotted line). The touchscreen interface provides concise and intuitive navigation information. Figure 4D includes several key elements: an orange border highlights the active area where the needle is being tracked and guided within the ultrasound image; a yellow dotted line indicates the projected path of the needle, helping to visualize the intended trajectory towards the target; a positioning point (×) marks the intersection of the puncture trajectory extension line and the ultrasound image plane, indicating the exact point of needle entry; an offset distance indicator shows the current distance between the tip of the needle and the ultrasound plane, divided into nine grids where the left four grids show the offset from the center to the front of the ultrasound image plane and the right four grids show the offset from the center to the back of the ultrasound image plane, ‘Front+’ indicates the direction of the cross product of *y*-axis and *x*-axis of ultrasound image and ‘Back-’ indicates the opposite direction of ‘Front+’, where *x*-axis is left to right and y-axis is top to bottom on ultrasound image; an offset angle indicator shows the angle between the current needle and the ultrasound image plane, divided into nine grids where the left four grids show the offset from the center to the front of the ultrasound image plane and the right four grids show the offset from the center to the back of the ultrasound image plane; a front view box displays the positioning information of the needle in the front view of the ultrasound probe, with the gray rectangle representing the ultrasound probe, the white straight line representing the ultrasound imaging plane, and the yellow dotted line indicating the projection of the puncture trajectory on a plane perpendicular to the ultrasound probe front view; and a tracking status box displays tracking status information and notification messages, ensuring the operator is informed about the system’s status and any necessary actions. These visual indicators assist the operator in accurately aligning the needle with the planned trajectory, ensuring precise and safe needle placement during the procedure.

**Figure 5:**
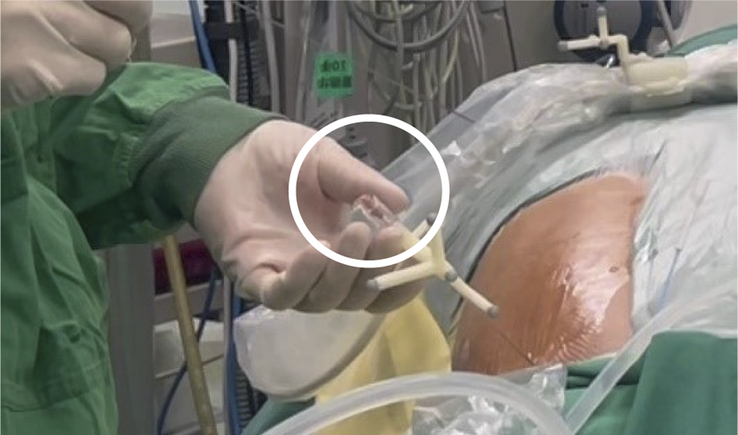
The puncture was confirmed by clear urine observed out of the needle cannula.

The general workflow of using the AcuSee system is described in Figure 3. Under general anesthesia, the patient is placed in the supine position for the insertion of a 5-Fr ureteral catheter; the patient is then repositioned to the prone position. Before the puncturing step, the ultrasound machine (model Arietta 60, HITACHI) is connected to the AcuSee system. Then tracking mounts are installed on the ultrasound probe (model C251, HITACHI) and the puncture needle (model 18G L-200MM, CREATEMEDIC). To select the ideal target calyx, an ultrasound scan is performed to observe the collecting system distended with saline injection through the ureteral catheter. A plan of an ideal needle trajectory is made, and the target calyx is punctured under the real-time guidance of the AcuSee system (Fig. 4). A successful puncture is precisely defined as the needle’s accurate placement within the targeted calyx, confirmed by the emanation of clear urine from the needle cannula, ensuring that the puncture is not only in the correct anatomical location but also functional for the procedure’s requirements (Fig. 5). Only ultrasound imaging and AcuSee guidance are used throughout the process, with no other imaging or guiding techniques. After a successful puncture, a standard minimally invasive PCNL procedure is performed.

The AcuSee system (Fig. 4D) display includes several key elements: an orange border highlights the active area where the needle is tracked and guided within the ultrasound image; a yellow dotted line indicates the projected path of the needle, helping to visualize the intended trajectory towards the target; a positioning point (×) marks the intersection of the puncture trajectory extension line and the ultrasound image plane, indicating the exact point of needle entry; an offset distance indicator shows the current distance between the tip of the needle and the ultrasound plane; an offset angle indicator shows the angle between the needle and the ultrasound image plane; and a tracking status box displays tracking status information and notification messages.

The guidance information is updated in real-time in the main task user interface (Fig. 3B-D). In the bottom left area, an image window displays the ultrasound image overlaid with the needle trajectory, including the part of the needle already within the image boundary (rectangular box) and its predicted trajectory (yellow dotted line). The top left area shows a front view of the needle trajectory (yellow dotted line) and the ultrasound image plane (white horizontal line) from the perspective of the probe head (gray rectangular box). The top right area contains the current selected settings and a control panel with interactive buttons. The bottom right area displays three spatial relationship indicators: the offset distance indicator showing the current distance between the needle tip and the ultrasound image plane, the offset angle indicator showing the angle between the needle trajectory and the ultrasound image plane, and the field-of-view (FOV) indicator showing the positions of the tracked probe (blue dot) and needle (yellow dot) within the FOV of the tracking module.

### CONSORT statement

This study adheres to the Consolidated Standards of Reporting Trials (CONSORT, Supplemental Digital Content 3, http://links.lww.com/JS9/D279) guidelines for reporting parallel group randomized trials, ensuring transparent, and complete reporting of this trial^29^.

### Statistical analysis

The demographic and clinical data of the participants were summarized using descriptive statistics. Continuous variables, such as age, BMI, CT values of stones, and stone size, were presented as mean values with ranges and compared using independent samples *t*-tests. Categorical variables, like sex, stone location, socioeconomic status, geographic distribution, smoking status, and alcohol consumption, were presented as frequencies and percentages. The stone clearance rate, complications, incidence of renal hydronephrosis, stone location, race, and medical history (including hypertension and diabetes) between the two groups were compared using the *χ* ^2^ test or Fisher’s exact test, as appropriate. The comparison of puncture attempts, puncture time, and total surgical time between the AcuSee guidance system group and the standard ultrasound-guided group was also conducted using independent samples *t*-tests. For the analysis of potential patient-specific factors affecting the performance of the AcuSee guidance system, linear regression was used to examine the correlation between BMI and puncture time, while the influence of renal morphology and prior surgical history on puncture time was assessed using the Mann–Whitney *U* test. For all analyses, a *P*-value of less than 0.05 (*P*<0.05) was considered statistically significant. All statistical analyses were performed using the SPSS version 21.0 software package (IBM).

## Results

### Study population

To evaluate the feasibility and safety of the AcuSee guidance system compared to standard ultrasound-guided PCNL, we conducted a randomized controlled trial with 58 patients (Table 1). Participants were randomly assigned to either the AcuSee guidance system group or the standard ultrasound-guided group, ensuring that any observed differences in outcomes could be attributed to the guidance system itself rather than pre-existing differences between patient groups.

**Table 1:** Demographic and clinical characteristics of the patients.

Characteristics	All (*N*=58)	AcuSee guidance system group (*N*=29)	Standard ultrasound-guided group (*N*=29)	*P*
Mean age, years (range)	55 (23–74)	55 (27–71)	55 (23–74)	0.92
Age Distribution (n, %)				0.49
18–30 years	2 (3.4)	1 (3.4)	1 (3.4)	
31–45 years	10 (17.2)	5 (17.2)	5 (17.2)	
46–60 years	28 (48.3)	16 (55.2)	12 (41.4)	
61+ years	18 (31.0)	7 (24.1)	11 (37.9)	
Sex, *n* (%)				1.00
Female	12 (20.7)	6 (20.7)	6 (20.7)	
Male	46 (79.3)	23 (79.3)	23 (79.3)	
Mean stone size, mm (range)	25.30 (12.00–46.70)	25.19 (12.00–45.00)	25.40 (12.40–46.70)	0.925
Hydronephrosis, *n* (%)				0.037
None	11 (19.0)	9 (31.0)	2 (6.9)	
Mild	30 (51.7)	10 (34.5)	20 (69.0)	
Moderate	13 (22.4)	8 (27.6)	5 (17.2)	
Severe	4 (6.9)	2 (6.9)	2 (6.9)	
Side, *n* (%)				0.424
Right	24 (41.4)	14 (48.3)	10 (34.5)	
Left	34 (58.6)	15 (51.7)	19 (65.5)	
Type of stones, *n* (%)				0.107
Pelvic	11 (19.0)	8 (27.6)	3 (10.3)	
Caliceal	2 (3.4)	2 (6.9)	0 (0.0)	
Pelvic and caliceal	38 (65.5)	15 (51.7)	23 (79.3)	
Staghorn	7 (12.1)	4 (13.8)	3 (10.3)	
CT value of stones, HU, (x±s)	1190.53±316.18	1127.47±326.76	1253.58±300.54	0.224
BMI, kg/m^2^, (x±s)	26.07±3.42	25.28±3.87	26.49±2.89	0.169
BMI, kg/m^2^, (*n*, %)				0.47
Underweight (<18.5 kg/m²)	1 (1.7)	1 (3.4)	0 (0.0)	
Normal weight (18.5–24.9 kg/m²)	20 (34.5)	12 (41.4)	8 (27.6)	
Overweight (25–29.9 kg/m²)	32 (55.2)	14 (48.3)	18 (62.1)	
Obese (≥30 kg/m²)	5 (8.6)	2 (6.9)	3 (10.3)	
Race (*n*, %)				0.69
Han Chinese	51 (87.9)	25 (86.2)	26 (89.7)	
Other ethnic groups	7 (12.1)	4 (13.8)	3 (10.3)	
Medical history (*n*, %)				
Hypertension	13 (22.4)	6 (20.7)	7 (24.1)	0.77
Diabetes	9 (15.5)	5 (17.2)	4 (13.8)	0.71
Prior kidney stone history	18 (31.0)	9 (31.0)	9 (31.0)	1.00
History of kidney stone-related surgeries	9 (15.5)	5 (17.2)	4 (13.8)	0.71
Socioeconomic status (*n*, %)				0.843
Low to lower-middle income (≤$4095)	14 (24.1)	8 (27.6)	8 (27.6)	
Upper-middle income ($4096–$12 695)	28 (48.3)	15 (51.7)	13 (44.8)	
High income (≥$12 696)	14 (24.1)	6 (20.7)	8 (27.6)	
Geographic distribution (*n*, %)				0.79
Northern China	34 (58.6)	18 (62.1)	16 (55.2)	
Southern China	24 (41.4)	11 (37.9)	13 (44.8)	
Smoking status (*n*, %)				0.77
Yes	13 (22.4)	7 (24.1)	6 (20.7)	
No	45 (77.6)	22 (75.9)	23 (79.3)	
Alcohol consumption (*n*, %)				0.57
Yes	18 (31.0)	10 (34.5)	8 (27.6)	
No	40 (69.0)	19 (65.5)	21 (72.4)	

CT, Computed Tomography; HU, Hounsfield Units.

The study included 46 males and 12 females, with a mean age of 55 years (range: 23–74 years). In the AcuSee group, the mean age was 55 years (range: 27–71 years), while in the standard group, it was also 55 years (range: 23–74 years), showing no significant difference in age distribution (*P*=0.92). We also assessed the BMI of participants. The overall mean BMI was 26.07 kg/m² (range: 17.15–35.49 kg/m²). The AcuSee group had a mean BMI of 25.28 kg/m² (range: 17.15–35.49 kg/m²), while the standard group had a mean BMI of 26.49 kg/m² (range: 20.50–32.90 kg/m²), with no significant difference between the groups (*P*=0.169).

Stone size and location were evaluated to understand their potential impact on the procedure. The overall mean stone size was 25.30 mm (range: 12.00–46.70 mm). In the AcuSee group, the mean stone size was 25.19 mm (range: 12.00–45.00 mm), and in the standard group, it was 25.40 mm (range: 12.40–46.70 mm), with no significant difference (*P*=0.925). Stone locations were also similar across groups, with the highest percentage located in both the renal pelvis and caliceal regions. In the AcuSee group, 51.7% of stones were in both regions, compared to 79.3% in the standard group (*P*=0.107). Additionally, we assessed the degree of hydronephrosis. Despite some differences in the severity of hydronephrosis (*P*=0.037), the overall comparability of the groups remained intact.

The CT values of the stones, which can indicate stone composition, were also compared. The mean CT value was 1190.53 HU overall, with 1127.47 HU in the AcuSee group and 1253.58 HU in the standard group, indicating no significant difference in stone composition between the groups (*P*=0.224). Both groups predominantly had nonuric acid stones.

Other demographic and clinical characteristics, such as sex distribution, side of stone occurrence, BMI categories, race, medical history (hypertension, diabetes, prior kidney stone history, history of kidney stone-related surgeries), socioeconomic status, geographic distribution, smoking status, and alcohol consumption, were similarly distributed between the groups, ensuring the homogeneity of the patient population.

In summary, the clinical data indicate that the two groups were homogeneous in their characteristics.

### Outcomes

#### Improved puncture efficiency with the AcuSee guidance system

**Figure 6:**
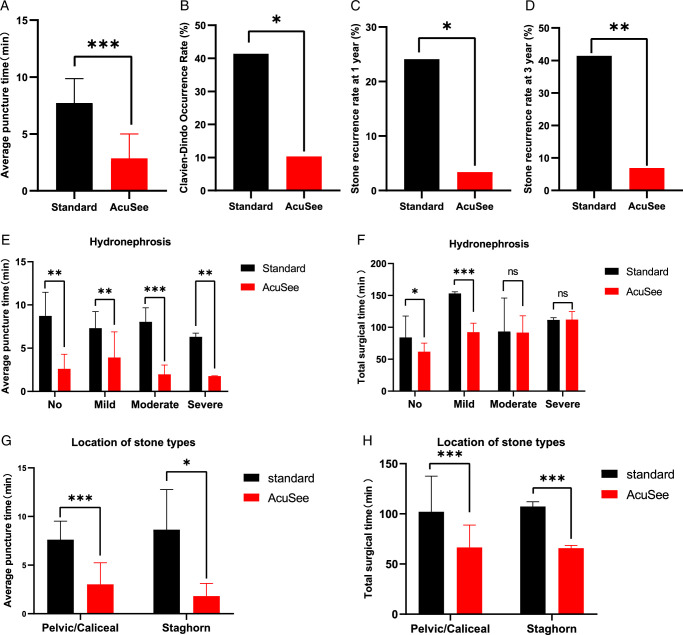
Comparison of clinical outcomes between the standard ultrasound-guided group and the AcuSee guidance system group in percutaneous nephrolithotomy (PCNL). (A) Average puncture time (minutes) was significantly shorter in the AcuSee group compared to the standard group. (B) The occurrence rate of Clavien–Dindo classified complications was lower in the AcuSee group. (C) The one-year stone recurrence rate (%) was significantly lower in the AcuSee group. (D) Three-years stone recurrence rate (%) was significantly lower in the AcuSee group. (E) Average puncture time (minutes) stratified by the degree of hydronephrosis (no, mild, moderate, and severe), showing significant reductions across all categories in the AcuSee group. (F) Total surgical time (minutes) stratified by the degree of hydronephrosis, demonstrating significant reductions in the no and mild hydronephrosis categories in the AcuSee group. (G) Average puncture time (minutes) stratified by location of stone types (renal pelvis/Caliceal, and Staghorn), showing significant reductions in the AcuSee group. (H) Total surgical time (minutes) stratified by location of stone types, demonstrating significant reductions in the AcuSee group. Statistical significance is indicated as follows: *P<0.05, **P<0.01, ***P<0.001, ****P<0.0001, ns, not significant.

The PCNL procedure was successfully completed for all the 58 patients (Table 2). The puncture accuracy, defined as the puncture success rate, was 100% for all the study patients. In terms of puncture efficiency, the average time taken from planning to successful puncture in the AcuSee guidance system group was 3.12 min (ranging from 0.2 to 6.88 min), demonstrating a significant 59% reduction compared to the 7.58 min (ranging from 5.41 to 10.68 min) observed in the standard ultrasound-guided group (Fig. 6A, Table 2). The degree of hydronephrosis can significantly affect the difficulty of the puncture procedure, with no hydronephrosis and mild hydronephrosis presenting greater challenges. Therefore, we conducted a subgroup analysis based on the degree of hydronephrosis to further evaluate the average puncture time and total surgical time (Fig. 6E, Table 2). For patients with no hydronephrosis, the average puncture time was 2.6±1.69 min in the AcuSee group and 8.73±2.75 min in the standard group (*P*=0.002), showing a 70% reduction. For mild hydronephrosis, the average puncture time was 4.27±3.83 min in the AcuSee group and 7.18±1.50 min in the standard group (*P*=0.005), indicating a 40% reduction. For moderate hydronephrosis, the average puncture time was 1.96±1.08 min in the AcuSee group and 8.08±1.58 min in the standard group (*P*<0.001), reflecting a 76% reduction. For severe hydronephrosis, the average puncture time was 1.78±0.35 min in the AcuSee group and 6.31±0.44 min in the standard group (*P*=0.005), representing a 71% reduction. Although the total surgical time is influenced by the time spent on anesthesia and lithotripsy, which may obscure the statistical effects of the puncture phase, the subgroup analysis results indicate that the AcuSee guidance system has a significant advantage in more challenging cases with no hydronephrosis and mild hydronephrosis (Fig. 6F, Table 2).

**Table 2:** Comparison of clinical outcomes between the AcuSee guidance system and standard ultrasound-guided groups.

Characteristics	All (*N*=58)	AcuSee guidance system group (*N*=29)	Standard ultrasound-guided group (*N*=29)	*P*
Average puncture time, minutes (x±s)				
No hydronephrosis	3.72±3.03	2.6±1.69	8.73±2.75	**0.002**
Mild hydronephrosis	4.50±3.41	4.27±3.83	7.18±1.50	**0.005**
Moderate hydronephrosis	5.93±2.43	1.96±1.08	8.08±1.58	**<0.001**
Severe hydronephrosis	4.04±2.63	1.78±0.35	6.31±0.44	**0.005**
Total surgical time, minutes (x±s)				
No hydronephrosis	76.63±30.26	61.70±13.55	84.70±33.68	**0.015**
Mild hydronephrosis	103.45±27.54	92.44±14.03	153.00±2.83	**<0.001**
Moderate hydronephrosis	92.38±36.45	91.75±26.41	93.40±52.58	0.950
Severe hydronephrosis	111.75±7.63	112.00±12.72	111.50±3.53	0.965
Average puncture time, minutes (x±s)				
Pelvic/Caliceal	5.36±3.09	3.02±2.22	7.62±1.90	**<0.001**
Staghorn	4.74±4.46	1.81±1.31	8.64±4.13	**0.024**
Total surgical time, minutes (x±s)				
Pelvic/Caliceal	84.68±34.47	66.56±22.24	102.12±35.47	**<0.001**
Staghorn	83.57±22.49	65.75±2.87	107.33±4.73	**<0.001**
Clavien–Dindo, n (%)				**0.015**
Grade I	8 (13.8)	2 (6.9)	6 (20.7)	
Grade II	4 (6.9)	1 (3.4)	3 (10.3)	
Grade III	3 (5.2)	0 (0)	3 (10.3)	
Grade IV	0 (0)	0 (0)	0 (0)	
Grade V	0 (0)	0 (0)	0 (0)	
Success rate of puncture in PCNL, *n* (%)	58 (100.0)	29 (100.0)	29 (100.0)	
Average puncture time, minutes (range)	5.35(0.2–10.68)	3.12 (0.2–6.88)	7.58 (5.41–10.68)	**<0.001**
Total surgical time, minutes (range)	87.67(29–200)	83 (29–122)	92.34 (38–200)	0.274
Stone clearance, *n* (%)	43 (74.1)	23 (79.3)	20 (69.0)	0.549
Median number of attempts	1	1	1	0.470
Stone recurrence rate at 1-year, *n* (%)	8 (13.8)	1 (3.4)	7 (24.1)	**0.05**
Stone recurrence rate at 3 years, *n* (%)	14 (24.1)	2 (6.9)	12 (41.4)	**0.004**

PCNL, percutaneous nephrolithotomy.

To further validate the advantages of the AcuSee guidance system, we conducted a subgroup analysis based on the location of stone types to evaluate the average puncture time and total surgical time (Fig. 6G and H, Table 2). For stones located in the renal pelvis/caliceal regions, the average puncture time was 3.02±2.22 min in the AcuSee group and 7.62±1.90 min in the standard group (*P*<0.001), showing a significant reduction. For staghorn stones, the average puncture time was 1.81±1.31 min in the AcuSee group and 8.64±4.13 min in the standard group (*P*=0.024), also demonstrating a significant reduction. In terms of total surgical time, for stones in the renal pelvis/caliceal regions, the AcuSee group required 66.56±22.24 min compared to 102.12±35.47 min in the standard group (*P*<0.001). For staghorn stones, the AcuSee group required 65.75±2.87 min compared to 107.33±4.73 min in the standard group (*P*<0.001), indicating significant reductions in both scenarios. These results further demonstrate the efficiency and effectiveness of the AcuSee guidance system in managing different types of kidney stones.

#### Analysis of puncture attempts

Next, we analyzed the number of puncture attempts. Among the 58 patients, 5 patients required 2 puncture attempts, and 1 patient required 3 puncture attempts. The basic case details are provided in Table 3.

**Table 3:** Detailed cases of multiple puncture attempts.

Case	Group	Sex	Age	BMI	Type of stones	Stone size (mm)	Side	Hydronephrosis	Total puncture time (min)	Postoperative complications	Number of attempts	Stone residue (mm)	Reasons for multiple puncture attempts
1	AcuSee guidance system group	Male	30	24.12	Pelvic and caliceal	40.2	Left	Mild	0.5	None	2	No	Significantly rotated kidney (patient-specific factor)
2	AcuSee guidance system group	Male	53	20.70	Staghorn	29.5	Right	None	2.73	None	2	No	Involuntary muscle spasms caused by anesthesia (patient-specific factor)
3	AcuSee guidance system group	Male	50	22.10	Pelvic and caliceal	26.3	Left	Mild	0.6	None	2	No	Significantly rotated kidney (patient-specific factor)
4	AcuSee guidance system group	Female	60	29.03	Staghorn	45	Right	None	3.33	None	3	No	Complex staghorn calculus and a large renal cyst near the target calyx (patient-specific factors)
5	Standard ultrasound-guided group	Female	23	20.5	Pelvic and caliceal	25.5	Left	Severe	6.62	DJ stent displacement	2	Yes, 5 mm	Severe tissue edema (limitation of ultrasound guidance)
6	Standard ultrasound-guided group	Male	63	23.8	Pelvic and caliceal	21.8	Right	Mild	7.18	None	2	No	Unclear anatomical landmarks on the two-dimensional ultrasound plane (limitation of ultrasound guidance)

In the standard ultrasound-guided group, out of the 29 patients, 27 punctures were successful on the first attempt. Two patients required two puncture attempts due to the limitations of ultrasound guidance. One patient had severe tissue edema, making it difficult to maintain the needle trajectory under ultrasound guidance (Case 5), and the other patient had unclear anatomical landmarks on the two-dimensional ultrasound plane, making it challenging to access the correct calyx (Case 6). Notably, one patient in the standard ultrasound-guided group (Case 5) had stone residue and postoperative complications including DJ stent displacement.

In the AcuSee guidance system group, out of the 29 patients, 25 punctures were successful on the first attempt. Three cases required two puncture attempts due to significantly rotated kidneys (Case 1 and Case 3) or involuntary muscle spasms caused by anesthesia during the procedure (Case 2). One case required three puncture attempts due to a complex staghorn calculus and a large renal cyst near the target calyx (Case 4). These challenges were not due to inadequacies of the AcuSee guidance system but were instead caused by patient-specific factors such as anatomical variations and physiological responses. Although the AcuSee group required 2–3 puncture attempts in some cases, the overall puncture time was significantly reduced compared to the standard ultrasound-guided group, and no postoperative complications or stone residues were observed in the AcuSee group. This highlights the efficiency and safety of the AcuSee guidance system. No significant difference in the number of puncture attempts was found between these two groups (*P*=0.470), reflecting the overall effectiveness of both guidance methods.

The findings indicate that the AcuSee guidance system effectively overcomes patient-specific challenges, maintaining a high level of efficiency and safety. For the AcuSee group, the failures were primarily due to patient-specific factors such as anatomical variations and involuntary muscle spasms. In contrast, the failures in the standard ultrasound-guided group were mainly due to the inherent limitations of ultrasound technology, such as difficulty in maintaining needle trajectory and unclear imaging. Discussing these failures and considering potential improvements for the AcuSee system, through incorporating adaptive algorithms to adjust for real-time anatomical changes, could further enhance the system’s performance in complex cases.

#### Influence of patient-specific factors on AcuSee system performance

To ensure a comprehensive evaluation of the AcuSee guidance system, we analyzed several patient-specific factors that could potentially influence its performance, including BMI, renal morphology, and prior surgical history. Firstly, we examined the correlation between BMI and puncture time. The data showed no significant correlation between BMI and puncture time (*P*=0.85), indicating that the performance of the AcuSee system is consistent across patients with varying body mass indices. Next, we evaluated the influence of renal morphology on the puncture time. This analysis considered anatomical variations and kidney positioning. The results showed no significant impact of renal morphology on puncture time (*P*=0.45), suggesting that the AcuSee system effectively accommodates different renal shapes and positions during the procedure. Lastly, we investigated the effect of prior surgical history on the puncture time. Patients with previous kidney surgeries might present additional challenges due to scar tissue and altered anatomy. However, our findings showed no significant difference in puncture time between patients with and without prior surgical history (*P*=0.77), indicating that the AcuSee system maintains its efficacy regardless of the patient’s surgical background.

These results indicate that patient-specific factors such as BMI, renal morphology, and prior surgical history do not significantly affect the performance of the AcuSee guidance system.

#### Stone clearance, safety, and complication rates with the AcuSee guidance system

The stone clearance rate for PCNL was 79.3% in the AcuSee group, which signifies a competent performance in managing kidney stones. Interestingly, no major intraoperative or postoperative complications (Grade III–V) were observed in the AcuSee guidance system group (Fig. 6B, Table 2). In contrast, the standard ultrasound-guided group faced notable complications: two patients required interventional embolization due to postoperative bleeding, and one patient encountered displacement of the DJ tube after surgery. These incidents represent Grade III complications according to the Clavien–Dindo classification, underscoring the potential risks associated with the standard procedure and highlighting the safety benefits of the AcuSee guidance system. Long-term effects were primarily assessed through the stone recurrence rate at 1-year and 3 years postoperation (Fig. 6C and D, Table 2). The stone recurrence rate at 1-year postoperation was significantly lower in the AcuSee group at 3.4% compared to 24.1% in the standard ultrasound-guided group. The stone recurrence rate at 3 years postoperation was also significantly lower in the AcuSee group at 6.9% compared to 41.4% in the standard ultrasound-guided group (*P*=0.004). This further demonstrates the long-term efficacy of the AcuSee guidance system in reducing the likelihood of stone recurrence.

#### Summary of efficacy and safety advantages of the AcuSee guidance system

Based on the above study results, we systematically summarize the outcomes. The AcuSee guidance system offers significant clinical advantages over standard ultrasound-guided methods in PCNL procedures. The system demonstrates a remarkable 59% reduction in average puncture time, enhancing procedural efficiency, particularly in patients with no or mild hydronephrosis. The AcuSee system consistently maintains high accuracy and safety, even in complex cases involving anatomical anomalies or physiological responses. Notably, the system’s effectiveness is consistent across diverse patient profiles, with no significant impact from factors such as BMI, renal morphology, or prior surgical history. Additionally, the AcuSee system achieves a higher stone clearance rate of 79.3%, with no major intraoperative or postoperative complications (Grade III–V) reported in the study group. This contrasts sharply with the notable complications observed in the standard group. Long-term efficacy is underscored by significantly lower stone recurrence rates at both 1-year and 3 years postoperation. The AcuSee system’s innovative integration of real-time imaging and user-friendly interface not only reduces puncture and surgical times but also enhances patient safety by avoiding major complications. Its economic efficiency, with a complete equipment cost of ~$6000 and a per-procedure cost of less than $50, reduces costs by about 50–70% compared to standard methods, making it a cost-effective solution for healthcare facilities. These savings improve resource allocation and accessibility of advanced kidney stone management. In summary, the AcuSee guidance system provides a safer, more efficient, and cost-effective alternative for PCNL, significantly improving patient outcomes and optimizing healthcare delivery.

## Discussion

This report is the first to describe the use of the AcuSee guidance system in a clinical setting to assist the puncture navigation technique for kidney access in patients undergoing PCNL. The present study successfully demonstrated the feasibility and safety of the AcuSee system. Accurate access to the renal collection system was successfully established for all 58 patients (100%). The mean puncture time starting from planning to completing a successful puncture was 3.12 min (0.2−6.88 min) in those who underwent PCNL using the AcuSee guidance system. The mean puncture time, starting from planning to completing a successful puncture, was 7.58 min (5.41–10.68 min) in the standard ultrasound-guided group. No surgical complications were observed during the procedure or during the postoperative follow-up, and neither the physicians nor patients received any radiation exposure during the entire PCNL procedure in the AcuSee guidance system group. Additionally, the complication rates were significantly lower in the AcuSee guidance system group compared to the standard ultrasound-guided group. No major intraoperative or postoperative complications (Clavien–Dindo Grade III–V) were reported in the AcuSee group, whereas the standard group experienced complications such as postoperative bleeding requiring interventional embolization and displacement of the DJ tube. The AcuSee system also demonstrated higher efficiency, with significantly reduced total surgical time, especially for patients with no and mild hydronephrosis. This reduction minimizes patient exposure to anesthesia and improves hospital workflow. Long-term outcomes further highlight the system’s efficacy, with a significantly lower 1-year stone recurrence rate in the AcuSee group (3.4%) compared to the standard group (24.1%). Additionally, the 3-year stone recurrence rate was also significantly lower in the AcuSee group (6.9%) compared to the standard group (41.4%). The AcuSee system also achieved a higher stone clearance rate of 79.3%, compared to the standard group’s lower clearance rate. This higher clearance rate is crucial for preventing future complications and improving patient quality of life postsurgery. Additionally, patient-specific factors such as BMI (*P*=0.85), renal morphology (*P*=0.45), and prior surgical history (*P*=0.77) did not significantly affect the performance of the AcuSee system, supporting its reliability and versatility in clinical practice. In summary, the AcuSee guidance system offers significant benefits over standard ultrasound guidance in PCNL procedures, including shorter puncture and surgical times, reduced complication rates, higher stone clearance rates, and lower stone recurrence rates. These benefits highlight the potential of the AcuSee system to enhance the safety, efficiency, and effectiveness of PCNL, ultimately leading to better patient outcomes and more efficient use of healthcare resources.

The establishment of percutaneous renal access relies on the support of imaging technology. Currently, the establishment of PCNL access by percutaneous renal puncture is mainly guided by radiographic or ultrasound imaging^30–34^. Radiographic imaging can clearly show the stone and kidney anatomy, which is conducive to percutaneous renal puncture. In addition, the guide wire placement and channel expansion can be monitored in real-time after a successful puncture, which improves the safety of channel establishment; however, the use of radiographic imaging inevitably results in radiation exposure to the patient and the operator^35–37^. Another disadvantage of radiography-guided PCNL is its poor imaging capability for soft tissues, such as the intestine, liver, pleura, and other organs surrounding the kidney, as well as the increased risk of related organ damage during percutaneous renal access establishment, which may lead to serious complications^38^.

With the advantages of real-time imaging, the ability to show the anatomic structures of the kidney and low cost, ultrasound guidance for PCNL is an effective method without ionizing radiation and is safe to use with pregnant patients^39–42^. AcuSee is an accessory to the ultrasound system and does not introduce any radiation risk throughout the procedure. However, the ultrasound image is a two-dimensional flat image with a narrow field of view and low resolution, which may provide extremely limited and nonintuitive information, as well as being difficult to operate and having a long learning curve^43^. Clinicians rely more on clinical experience to determine the relative positions between the ultrasound probe, the puncture needle, and the target site, and they use this expertise to plan the puncture path. Because of the patient’s breathing, tissue deformation, and other uncertainties during the puncture procedure, medical imaging-based puncture navigation systems require online planning based on the dynamic environment of the procedure and the current puncture status to accurately puncture the target site of the lesion^44–46^. For all of these reasons, ultrasound-guided PCNL is technically more challenging, especially for the treatment of patients with complex renal stones, such as partial or complete staghorn calculi in the renal pelvis or calyces^47,48^. Such procedures may have greater technical difficulty and a higher risk of complications; therefore, technical modifications are required to improve their safety and efficacy. Table 4 shows the advantages and disadvantages of the AcuSee guidance system and the standard ultrasound guidance method.

**Table 4:** Advantages and disadvantages of the AcuSee guidance system and the standard ultrasound guidance method.

Methods	AcuSee guidance system	Standard ultrasound-guided method
Advantages	(1) Radiation-free	(1) Low cost;
	(2) Capability of working with any existing ultrasound machine and puncture needle;	(2) Without ionizing radiation and is safe to use with pregnant patients;
	(3) Accurate real-time guidance on needle trajectory;	
	(4) Visualization of the spatial relationship between the needle and ultrasound plane;	
	(5) Large tracking volume which allows both in-plane and out-of-plane needle puncture;	
	(6) No risk from in vivo use of any new device;	
	(7) Convenient setup, minimal interference with operations and intuitive interaction.	
	(8) Low cost;	
Disadvantages	(1) The operator must keep tracking mounts visible to the camera to make tracking work;	(1) A two-dimensional flat image with a narrow field of view and low resolution;
	(2) The guidance accuracy may be impaired when the needle is bent.	(2) Difficult to operate and having a long learning curve;
		(3) Rely more on clinical experience to determine the relative positions between the ultrasound probe, the puncture needle, the target site, and the puncture path;
		(4) Patient’s breathing, tissue deformation, and other uncertainties during the puncture procedure will affect the accuracy of puncture

In response to these challenges, some newer surgical navigation techniques have been developed and applied to PCNL procedures. The SonixGPS, based on an electromagnetic tracking navigation system, has been used for complex operations such as renal puncture navigation, nerve blocks, and vascular access^16–18,49^. Its greatest advantage is that the operator can consistently track the needle position, even if the needle position is not in the same plane as the ultrasound image in the deep tissue, improving the safety of the operation and reducing the operation time. However, this positioning system has the disadvantages of the requirement of in vivo use of additional transducers (installed inside the needle) and serious accuracy degradation due to electromagnetic interference from other devices in the operating room^17,19^. In addition, it requires the purchase of a new high-cost ultrasound device. On the other hand, AcuSee can work with any ultrasound machine and puncture needle that are already available, which means much less financial cost for healthcare providers. Rassweiler *et al*. described an iPad-assisted renal puncture in which the intraoperative patient images on the iPad are transmitted via a wireless network and then fused with virtual preoperative three-dimensional CT images^50,51^. This approach has two advantages: 1) 3D images clearly show the anatomy of the organ and its adjacent organs, thus reducing spatial errors; 2), this strategy reduces the training cycle for young surgeons. However, its disadvantages include exposure to ionizing radiation, no real-time 3D images and a less adjustable range of puncture paths. AcuSee, on the contrary, gives real-time guidance information and lets users operate the needle more freely with its wide field of view.

Hamamoto *et al*.^20^ explored a real-time, virtual, ultrasound-guided technique for percutaneous renal puncture. Preoperatively, the patient’s prone enhanced CT images were automatically reconstructed as multiplanar reconstructed images of the kidney by software, and then the ultrasound images were synchronised to the multiplanar reconstructed images of the kidney for fusion so that the two images overlap simultaneously. A percutaneous renal puncture was performed under the guidance of the reconstructed fusion images and adjusted to obtain an accurate puncture under the direct view of the fibreoptic ureteroscope. Satisfactory results were obtained in the puncture of 15 patients compared with those undergoing procedures guided by conventional ultrasound. However, the disadvantages are that the technique is complicated for operators, and the processing of the images requires specialised software. In contrast, AcuSee provides a touchscreen interface that can be activated with a single click, with concise and intuitive navigation information, and does not require complex preoperative preparation.

Based on optical tracking and augmented reality technologies, the AcuSee guidance system enables visualization of the needle trajectory throughout the process of needle puncture during PCNL. With live ultrasound image guidance, the surgeon can easily monitor the needle tip position, the needle trajectory orientation, and the spatial relationship between the needle and the ultrasound image plane in terms of the offset angle and offset distance between the needle trajectory and the ultrasound image plane. When ultrasound needle visualization is unavailable under ultrasound, no matter before the needle is placed under the skin or when the needle is out of the ultrasound image plane, this method still allows the surgeon to track the needle trajectory and align it with the puncture target. If necessary, the guidance information can also help the surgeon to realign the needle to be coplanar with the image plane. This technique has several advantages: 1) radiation-free throughout the procedure; 2) capability of working with any existing ultrasound machine and puncture needle; 3) accurate real-time guidance on needle trajectory; 4) visualization of the spatial relationship between the needle and ultrasound plane; 5) large tracking volume which allows both in-plane and out-of-plane needle puncture; 6) no risk from in vivo use of any new device (the tracking mounts are held outside the patient); 7) convenient setup, minimal interference with operations and intuitive interaction; 8) Low cost, a complete set of equipment requires only $6000, with the cost per patient per-procedure being less than $50. This economic efficiency makes the AcuSee guidance system an affordable option for many healthcare facilities, potentially increasing its accessibility and widespread adoption in the field of minimally invasive PCNL procedures. Moreover, this technique has the potential to improve the accuracy and efficiency of expert surgeons and help shorten the learning curve for beginners. However, some disadvantages of this technique have also been discovered: 1) the operator must keep tracking mounts visible to the camera to make tracking work and 2) the guidance accuracy may be impaired when the needle is bent.

While the AcuSee guidance system has demonstrated significant benefits in terms of efficiency, accuracy, and safety, it is essential to critically evaluate its limitations. The system’s performance in complex cases, such as those involving patients with anatomical anomalies or significant renal deformities, requires further investigation. Extreme cases of hydronephrosis or highly rotated kidneys could potentially impact the accuracy of needle trajectory guidance. Future studies should aim to address these challenges by incorporating adaptive algorithms that can adjust for real-time anatomical changes and physiological responses during the procedure. Potential enhancements to the AcuSee system include integration with other imaging technologies such as CT or MRI, which could provide a more comprehensive view of the renal anatomy and surrounding structures. Advancements in augmented reality could further enhance visualization, offering surgeons even more precise guidance during complex PCNL procedures. This could potentially minimize the learning curve for novice surgeons and improve overall surgical outcomes. The AcuSee system presents several advantages that can help mitigate potential barriers such as cost, training requirements, and regulatory hurdles. The initial investment in the AcuSee system is relatively low, with the complete equipment costing ~$6000 and a per-procedure cost of less than $50. Compared to standard methods, costs can be reduced by about 50–70%^6^. These savings can alleviate the financial burden on healthcare facilities and improve resource allocation. Furthermore, the AcuSee system’s straightforward integration with existing ultrasound machines and its user-friendly interface can reduce the training time required for surgeons, making it easier to implement in various clinical settings. Addressing regulatory hurdles will be crucial for its adoption, but the demonstrated safety and efficacy in our study provide a strong foundation for obtaining necessary approvals. Clear documentation of the system’s benefits and cost-effectiveness can support its case for regulatory approval and encourage broader adoption.

We recognize that this study has some limitations. First, we did not enroll patients with body mass index >36 kg/m^2^ because of the difficulty with ultrasound imaging of the kidneys of patients with severe obesity or those with ectopic kidneys. Second, because of the small number of study participants, the results may be biased. Our future investigations will include more patients and use a multicentre RCT design.

## Conclusion

Our RCT study demonstrates that the AcuSee guidance system significantly improves procedural efficiency, accuracy, and safety in PCNL compared to the standard ultrasound-guided method. The system achieves a remarkable 59% reduction in puncture time, maintains high accuracy even in complex cases, and consistently performs well across diverse patient profiles. Additionally, it shows a higher stone clearance rate and significantly lower complication and recurrence rates. To further validate these findings, future research should focus on larger-scale clinical trials and multicenter studies. Long-term patient outcomes and comprehensive cost-benefit analyses are essential to establish the lasting benefits and economic impact of the AcuSee system. Exploring its potential applications in other surgical and medical procedures could further enhance its clinical value and utility.

## Ethical approval

Informed consent was obtained from all eligible participants before the procedure. Institutional review board approval (2020keyan398) was obtained from PKUFH before starting the study.

## Consent

Written informed consent was obtained from the patient for publication of this case report and accompanying images. A copy of the written consent is available for review by the Editor-in-Chief of this journal on request.

## Source of funding

This study was funded by National Key R&D Program of China(2023YFC2415500), 2023 Shenzhen Second People's Hospital Clinical Research Program (2023yjlcyj016), National Natural Science Foundation of China (No. 82273135), Beijing Municipal Science & Technology Commission (No. Z221100007422073), 2023 Beijing Municipal Health Commission Capital Medical Science and Technology Innovation Achievement Transformation Excellent Promotion Program Project (YC202301QX0162), National High Level Hospital Clinical Research Funding (Scientific and Technological Achievements Transformation Incubation Guidance Fund Project of Peking University First Hospital, 2024CX24), National High Level Hospital Clinical Research Funding (Peking University First Hospital Domestic Multicenter Clinical Research Special Fund, 2022CR54), National High Level Hospital Clinical Research Funding (Peking University Medical Innovation Translation Special Fund, 2022FY03), National High Level Hospital Clinical Research Funding (Peking University First Hospital Scientific and Technological Achievement Transformation Incubation Guidance Fund Project, 2022CX02).

## Author contribution

A.L., P.W., Y.X., and L.Y.: designed this work; C.X., A.L., P.W., Y.X., and L.Y.: integrated and analyzed the data; C.X., L.L., and G.X.: wrote this manuscript; X.L., Y.F., Z.Z., X.L., and X.Z.: edited and revised the manuscript. All authors approved this manuscript.

## Conflicts of interest disclosure

The authors declare that they have no competing interests.

## Research registration unique identifying number (UIN)

1. Name of the registry: Chinese Clinical Trial Registry.
2. Unique identifying number or registration ID: ChiCTR2300078988.
3. Hyperlink to your specific registration (must be publicly accessible and will be checked): https://www.chictr.org.cn/.

## Guarantor

The Guarantor for this study is Dr Lin Yao. Dr Yao accepts full responsibility for the work as reported and for the conduct of the study. He had full access to all the data in the study and takes responsibility for the integrity of the data and the accuracy of the data analysis. Dr. Yao controlled the decision to publish and is prepared to handle questions and policy issues regarding the data. Dr. Yao affirms that all aspects of the study, including its data integrity and adherence to reporting protocols, are transparent and honest.

## Data availability statement

The datasets generated during and/or analyzed during the current study are not publicly available but are available from the corresponding author on reasonable request and with the author’s permission. The data are subject to privacy and ethical restrictions aimed at protecting the confidentiality and rights of participants involved in the study.

## Provenance and peer review

This paper was not invited; it was submitted independently by the authors.

## Supplementary Material
